# The Klingon batbugs: Morphological adaptations in the primitive bat bugs, *Bucimex chilensis *and *Primicimex cavernis*, including updated phylogeny of Cimicidae

**DOI:** 10.1002/ece3.4846

**Published:** 2019-01-24

**Authors:** Gonzalo Ossa, Joseph S. Johnson, Anna I. E. Puisto, Veikko Rinne, Ilari E. Sääksjärvi, Austin Waag, Eero J. Vesterinen, Thomas M. Lilley

**Affiliations:** ^1^ ConserBat EIRL San Fabian Chile; ^2^ Department of Biological Sciences Ohio University Athens Ohio; ^3^ Biodiversity Unit University of Turku Turku Finland; ^4^ Department of Agricultural Sciences University of Helsinki Helsinki Finland; ^5^ Institute of Integrative Biology University of Liverpool Liverpool UK; ^6^ Finnish Museum of Natural History University of Helsinki Helsinki Finland

**Keywords:** Chiroptera, Cimicinae, dispersal, ectoparasite, tarsal structure

## Abstract

The Cimicidae is a family of blood‐dependent ectoparasites in which dispersion capacity is greatly associated with host movements. Bats are the ancestral and most prevalent hosts for cimicids. Cimicids have a worldwide distribution matching that of their hosts, but the global classification is incomplete, especially for species outside the most common Cimicidae taxa. In this study, we place a little‐studied cimicid species, *Bucimex chilensis*, within a comprehensive molecular phylogeny of Cimicidae by sequencing the genomic regions of this and other closely related species. For this study, we collected *B. chilensis *females from *Myotis chiloensis* in Tierra del Fuego, 1,300 km further south than previously known southernmost distribution boundary. We also sequenced COI regions from *Primicimex cavernis*, a species which together with *B. chilensis* comprise the entire subfamily Primiciminae. Using Bayesian posterior probability and maximum‐likelihood approaches, we found that *B. chilensis* and *P. cavernis* clustered close to each other in the molecular analyses, receiving support from similar morphological features, agreeing with the morphology‐based taxonomic placement of the two species within the subfamily Primiciminae. We also describe a previously unrecognized morphological adaptation of the tarsal structure, which allows the austral bat ectoparasite, *B. chilensis*, to cling on to the pelage of its known host, the Chilean myotis (*Myotis chiloensis*). Through a morphological study and behavioral observation, we elucidate how this tarsal structure operates, and we hypothesize that by clinging in the host pelage, *B. chilensis* is able to disperse effectively to new areas despite low host density. This is a unique feature shared by *P. cavernis*, the only other species in Primiciminae.

## 1 INTRODUCTION

Parasitism is a widespread lifestyle, with parasitic organisms found in many taxa and constituting as much as 50% of animal biodiversity (Poulin & Morand, 2000; Weinstein & Kuris, 2016). Thus, parasites are important not only for their notable interactions with their hosts, but also evolutionarily, as they provide opportunities to test numerous hypotheses on speciation (Morand & Poulin, 2003). Constructing parasite phylogenies using molecular methods has opened the door for research in this area, as well as providing a broader understanding of relationships among parasites and their hosts. Therefore, phylogenies of diverse or widespread groups of parasites are useful in studies of parasite speciation, or coevolution of parasites and their hosts (Hafner & Nadler, 1988).

The Cimicidae (Heteroptera) are an ecologically important family of parasites with a phylogeny which could benefit from more attention despite recent advances made by Balvín (2015), Balvín, Munclinger, Munclinger, Kratochvíl, and Vilímová (2012). Cimicids are obligate hematophagous ectoparasites that are distributed across the globe and contain 110 described species within 24 genera in six subfamilies (Henry, 2009). Both sexes feed exclusively on blood, and development into a subsequent instar, as well as egg production in adult females and sperm production in males requires a blood meal (Reinhardt & Siva‐Jothy, 2007; Waage, 1979). Cimicids are proposed to have evolved from predatory heteropteran ancestors, but roughly 60% of extant cimicid species specialize on parasitizing bats (Poulin & Morand, 2000) (Chiroptera). Bats are considered the ancestral hosts of cimicids, although humans and other vertebrates may be used as secondary hosts (Hornok et al., 2017; Usinger, 1966).

Bats, the second largest mammalian order, are highly social animals (Kerth, 2008). During pregnancy and lactation, many bat species establish maternity colonies in roosts with relatively stable climatic conditions to give birth to their young. Both the bats and their roosts provide a suitable environment for arthropod ectoparasites (Lucan, 2006), but the social behaviors of bats also represents risks to these parasites. Social grooming has been observed in a number of bat species, which exposes these parasites to other members of the social group of hosts (Kerth, Almasi, Ribi, Thiel, & Lüpold, 2003). However, it is difficult for bats to protect themselves by grooming against cimicids. Cimicids are able produce a defense substance and bats refuse to bite them (Usinger, 1966), although it is not completely unlikely for this to occur (Bartonicka, 2008). Generally, adult cimicids feed in periods of few days, and only while bats are normothermic (Bartonicka, 2008), after which most species leave the immediate vicinity of the host to digest the meal within the confines of the roost and can survive up to a 1.5 years without feeding again (Johnson, 1941). The latter allows them to overwinter at summer roosts even after bats have migrated to hibernation sites. The ability to survive long periods without meals may be an especially important adaptive trait in Cimicidae, which appear to have a low inherent capacity for dispersal over long distances, and even short distance movements seem to be limited (Talbot, Vonhof, Broders, Fenton, & Keyghobadi, 2016; Usinger, 1966). In fact, without the ability to fly, it is unlikely that adult cimicids are able to disperse without the host (Balvín, Sevcik, et al., 2012; Brown & Bomberger‐Brown, 1996; Usinger, 1966). Although the phylogenetic topology between cimicids and their specific bat hosts have not been studied in great detail, the biology and ecology of many cimicids appears to be strongly influenced by the host species and their ecology (Balvin, Bartonicka, Simov, Paunovic, & Vilimova, 2014; Balvín, Munclinger, et al., 2012; Hafner & Nadler, 1988).

Inseminated cimicid females are observed attached to forearms of bats outside roosts more often than males or non‐inseminated females, suggesting cimicids primarily travel on bats to disperse (Balvín, Sevcik, et al., 2012; Heise, 1988). However, transmission appears to be uncommon (Talbot et al., 2016), possibly because this mode of dispersal poses inherent risks to cimicids, as cimicids lack morphological adaptations to properly attach to the host for prolonged periods of time and are easily discarded during grooming. Thus, although cimicids hosts are highly mobile, cimicid populations may be more genetically isolated than those of their hosts (Talbot et al., 2016). Unfortunately, cimicids spend most of their lives in cryptic bat roosts and are therefore seldom available for study. Although the European fauna of cimicids are well described, numerous gaps remain in global cimicid taxonomy, host specificity and ecology from other continents. Filling these gaps will provide opportunities to test novel hypotheses on the ecology and evolution of these unique ectoparasites of bats, which being the only flying mammals, are highly mobile and distributed across the globe.

Until recently, the only phylogeny of Heteroptera was built exclusively on morphology (Usinger, 1966). This phylogeny, and the positioning of Cimicomorpha within Heteroptera, exhibited a number of inconsistencies compared to molecular data presented by Balvín et al.(2015), which however, concentrated on the genus Cimex, rather than the family as a whole. More recent molecular phylogenies add to this (Hornok et al., 2017), but besides a hypothetical phylogeny by Reinhardt and Siva‐Jothy (Reinhardt & Siva‐Jothy, 2007), they do not provide a comprehensive description of Cimicomorpha, or Cimicidae (Li, Tian, Zhao, & Bu, 2012; Schuh, Weirauch, & Wheeler, 2009). This was rectified by Balvín et al. (2015), but even they did not include some of the sister groups outside the four common Cimicidae species groups within the genus Cimex (*Cimex lectularius* L.*, Cimex pilosellus *(Horvath, 1910)*, Cimex hemipterus* (Fabricius, 1803) and *Cimex pipistrelli *(Jenyns, 1839).

Herein we describe novel morphological adaptations in the tarsal structure of the bat ectoparastite, *Bucimex chilensis* Usinger, 1963, which may allow for its more effective dispersal. We compare the morphology of *B. chilensis* to its closest known relative, *Primicimex cavernis* Barber, 1941, which shares many of the same distinguishable features (Usinger, 1966). These two species are the only known described taxa of the subfamily Primicimicinae, and are classified in monotypic genera (Usinger, 1966). Both species are associated solely with bats in the western hemisphere. Using DNA samples from both species, we add to the phylogeny of the family *Cimicidae* using both nuclear and mitochondrial sequence data as well as describe a new geographic record for *B. chilensis*.

## 2 MATERIAL AND METHODS

### 2.1 Bat capture methods and location

**Figure 1:**
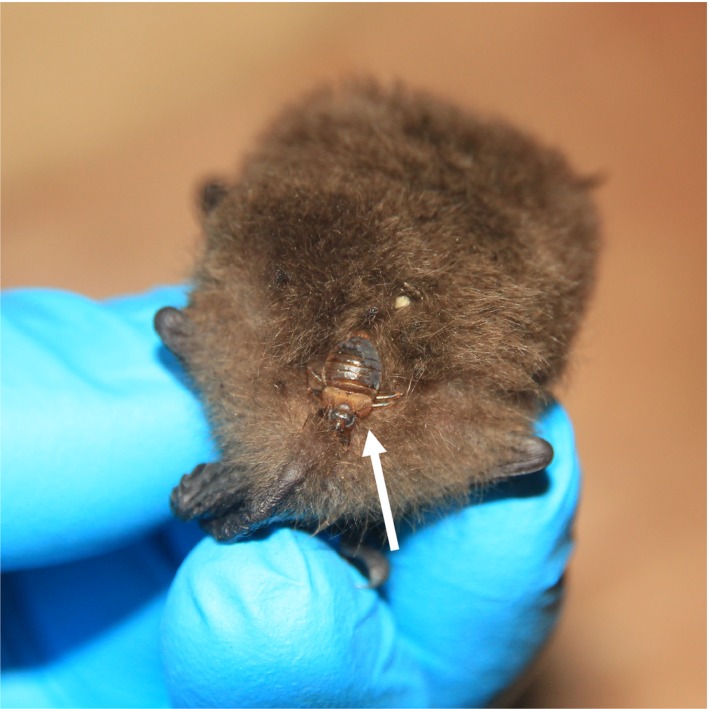
*Bucimex chilensis* (white arrow) at the base of the tail, on the dorsal surface of *Myotis chiloensis*

We captured two adult female *Myotis chiloensis* (Waterhouse, 1840, Capture permit #1253‐2016 by the Servicio Agrícola y Ganadero, Chile [SAG]) at Karukinka Reserve in southern Tierra del Fuego (54°S, 69°W; elevation 159 meters above sea level) with *B. chilensis* females attached to the pelages on the dorsal surfaces (Figure 1). The captures were from two consecutive years, November 2016 and December 2017. This capture site is located 1,300 km south of the previously described southernmost distribution of *B. chilensis *(Usinger, 1966, Figure 2)*.* The Karukinka reserve is situated in the sub‐antarctic phytogeographic province, with precipitation between 450 and 1,100 mm/year, and a mean annual temperature of 7°C (Arroyo et al., 1995). The habitat surrounding the capture site is dominated by *Nothofagus pumilio *(Poepp. & Endl.) Krasser, a deciduous tree species, mixed with *Nothofagus betuloides *(Mirb.) Oerst., an evergreen tree species (Arroyo et al., 1995) and a high diversity of mosses and lichens (Armestó, Villagrán, & Kalin Arroyo, 1996). In addition to the *B. chilensis* from Tierra del Fuego, two individuals of *P. cavernis* were obtained from the Smithsonian Institution National Museum of Natural History USA, for photography and sequencing. These specimens were collected in Ney Cave in Medina County, Texas, USA. We collected samples from *Cimex pilosellus* and *C. adjunctus* from Manitoba, Canada. Additionally, we received samples for *C. lectularius* from various locations in Finland, including regions of Turku, Tampere, Oravainen, and Kemiö. See Table 1 for details all samples in this study.

**Figure 2:**
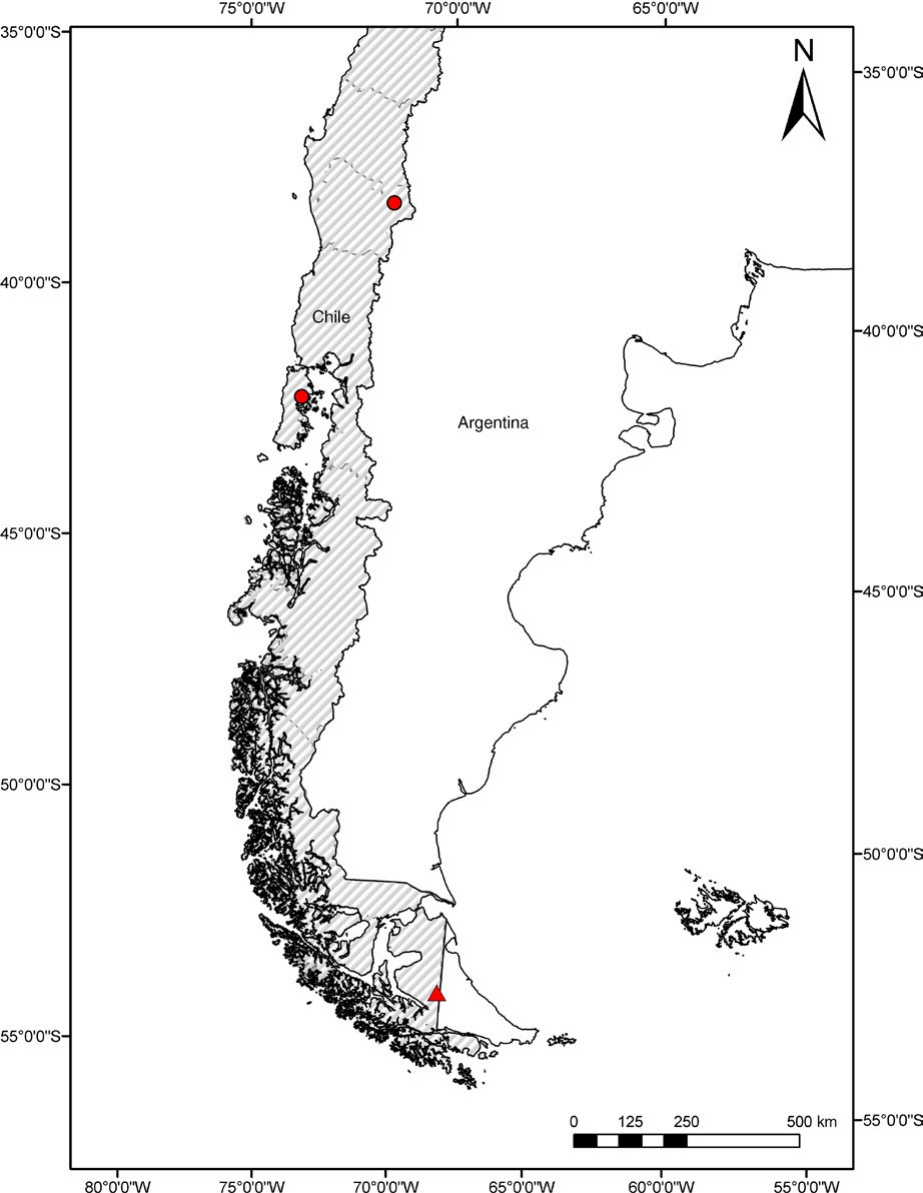
Map of austral South America. Previous collection sites of *Bucimex chilensis* are indicated with red dots. The present sample collection site is indicated with a red triangle

**Table 1:** Samples used in study and accession numbers for sequences (Ossa et al.)

Sample ID	Species	Collection country	References	ITS2	16S	EF1	COI	18S
KT380159*	*Leptocimex inordinatus*	Thailand	Potiwat, Sungvornyothin, Samung, Payakkapol, and Apiwathnasorn (2016)	–	–	–	KT380159	–
KT380160*	*Leptocimex inordinatus*	Thailand	Potiwat et al. (2016)	–	–	–	KT380160	–
KT380161*	*Leptocimex inordinatus*	Thailand	Potiwat et al. (2016)	–	–	–	KT380161	–
Pcav‐1*	*Primicimex cavernis*	USA	This study	–	–	–	MK141690	–
Buci‐1	*Bucimex chilensis*	Chile	This study	MK205317	MK190909	–	MK141702	MK201662
Buci‐2	*Bucimex chilensis*	Chile	This study	MK205318	MK190910	–	MK141694	MK201663
BB‐2	*Cimex pilosellus*	Canada	This study	MK205330	MK190908	MK213763	MK141693	MK201659
BB‐9	*Cimex adjunctus*	Canada	This study	MK205332	‐	‐	MK141701	MK201660
BB‐4	*Cimex adjunctus*	Canada	This study	MK205331	‐	‐	MK141691	MK201661
ORA‐2	*Cimex lectularius*	Finland	This study	MK205323	‐	‐	MK141703	MK201664
TKU‐8	*Cimex lectularius*	Finland	This study	MK205325	‐	‐	MK141706	MK201667
TKU‐5	*Cimex lectularius*	Finland	This study	MK205322	‐	‐	MK141704	MK201665
TKU‐6	*Cimex lectularius*	Finland	This study	MK205327	‐	‐	MK141705	MK201666
TRE‐1	*Cimex lectularius*	Finland	This study	MK205326	‐	MK213764	MK141695	MK201668
WE‐1	*Cimex lectularius*	Finland	This study	MK205319	‐	MK213765	MK141696	MK201669
WE‐2	*Cimex lectularius*	Finland	This study	MK205328	‐	MK213766	MK141692	MK201670
WE‐3	*Cimex lectularius*	Finland	This study	MK205324	‐	MK213767	MK141697	MK201671
WE‐4	*Cimex lectularius*	Finland	This study	MK205329	‐	MK213768	MK141698	MK201672
WE‐5	*Cimex lectularius*	Finland	This study	MK205320	‐	MK213769	MK141699	MK201673
WE‐6	*Cimex lectularius*	Finland	This study	MK205321	‐	MK213770	MK141700	MK201674
48	*Cimex pipistrelli group 2*	Czech Rep.	Balvín et al. (2015)	KC503542	GU985553	KC503545	GU985531	KC503546
52	*Cimex pipistrelli group 1*	Czech Rep.	Balvín et al. (2015)	KC503543	GU985549	KC503545	GU985527	KC503547
57	*Cimex pipistrelli group 2*	Czech Rep.	Balvín et al. (2015)	KC503542	GU985555	KC503545	GU985533	KC503548
61	*Cimex pipistrelli group 2*	Czech Rep.	Balvín et al. (2015)	KC503543	GU985551	KC503545	GU985529	KC503549
62	*Cimex pipistrelli group 2*	Czech Rep.	Balvín et al. (2015)	KC503542	GU985554	KC503545	GU985531	KC503550
73	*Cimex pipistrelli group 1*	Czech Rep.	Balvín et al. (2015)	KC503542	GU985550	KC503545	GU985528	KC503551
83	*Cimex pipistrelli group 2*	U.K.	Balvín et al. (2015)	KC503543	GU985556	KC503545	GU985534	KC503552
103	*Cimex pipistrelli group 2*	Bulgaria	Balvín et al. (2015)	KC503542	GU985552	KC503545	GU985530	KC503553
350	*Cimex pipistrelli group: Cimex japonicus*	Japan	Balvín et al. (2015)	KF018700	KF018727	KF018744	KC503541	KF018713
140	*Cimex adjunctus (C. pilosellus group)*	USA	Balvín et al. (2015)	KF018699	GU985558	KF018742	GU985536	KF018712
141	*Cimex adjunctus (C. pilosellus group)*	USA	Balvín et al. (2015)	KF018698	GU985557	KF018741	GU985535	KF018712
142	*Cimex adjunctus (C. pilosellus group)*	USA	Balvín et al. (2015)	KF018699	GU985559	KF018743	GU985537	KF018712
TM_C10	*Cimex cf. antennatus (C. pilosellus group)*	USA	Balvín et al. (2015)	KF018705	KF018732	KF018749	KF018760	KF018718
KR_C18	*Cimex latipennis (C. pilosellus group)*	Canada	Balvín et al. (2015)	KF018707	KF018734	KF018750	KF018758	KF018720
KR_C19	*Cimex latipennis (C. pilosellus group)*	Canada	Balvín et al. (2015)	KF018706	KF018733	KF018750	KF018757	KF018719
KR_C20	*Cimex pilosellus*	USA/Canada	Balvín et al. (2015)	KF018704	KF018731	KF018748	KF018759	KF018717
145	*Cimex hemipterus*	Malaysia	Balvín et al. (2015)	KF018695	KF018724	KF018710	KF018754	KF018739
801	*Cimex hemipterus*	India	Balvín et al. (2015)	KF018696	KF018725	KF018710	KF018755	KF018739
26	*Cimex lectularius*	Czech Rep.	Balvín et al. (2015)	KF018697	GU985546	KF018740	GU985524	KF018711
39	*Cimex lectularius*	Czech Rep.	Balvín et al. (2015)	KF018697	GU985548	KF018740	GU985526	KF018711
46	*Cimex lectularius*	Czech Rep.	Balvín et al. (2015)	KF018697	GU985547	KF018740	GU985525	KF018711
110	*Cimex lectularius*	France	Balvín et al. (2015)	KF018697	GU985545	KF018740	GU985523	KF018711
133	*Cimex lectularius*	Serbia	Balvín et al. (2015)	KF018697	KF018726	KF018740	KF018756	KF018711
120	*Oeciacus hirundinis*	Czech Rep.	Balvín et al. (2015)	KF018691	GU985565	KF018736	GU985543	KF148594
130	*Oeciacus hirundinis*	Germany	Balvín et al. (2015)	KF018692	GU985567	KF018736	GU985544	KF148594
149	*Oeciacus vicarius*	USA	Balvín et al. (2015)	KF018694	GU985563	KF018738	GU985541	KF018709
KR_88‐10n1	*Oeciacus vicarius*	USA	Balvín et al. (2015)	KF018694	KF018723	KF018738	KF018753	KF018709
KR_88‐10n3	*Oeciacus vicarius*	USA	Balvín et al. (2015)	KF018694	KF018722	KF018738	KF018752	KF018709
897	*Cimex sp.*	Japan	Balvín et al. (2015)	KF018693	GU985564	KF018737	GU985542	KF018708
C9	*Paracimex setosus*	–	Balvín et al. (2015)	–	KF018735	KF018751	KF018761	KF018721
240	*Cacodmus vicinus*	Jordan	Balvín et al. (2015)	KF018701	KF018728	KF018745	KF018762	KF018714
244	*Cacodminae sp.*	Mauretania	Balvín et al. (2015)	KF018702	KF018730	KF018747	KF018764	KF018716
243	*Aphrania elongata*	Mauretania	Balvín et al. (2015)	KF018703	KF018729	KF018746	KF018763	KF018715
ONI	*Anthocoridae: Orius niger*	–	Hua et al. (2008); Jung et al. (2010)	–	NC012429	–	NC012429	GQ258418.1
LEL	*Miridae: Lygus elisus*	–	Wheeler & Shuh (unpubl.)	–	AY252785.1	–	HM215068.1	AY252310.1
RPR	*Reduviidae: Rhodnius prolixus*	–	Gaunt & Miles (2002); García, Manfredi, Fichera, and Segura (2003)		AF324519.1, EU822954.1	ACPB02032738.1	AF449138.1	AY345868.1

* Only included in the data set 2.

### 2.2 Digital layer imaging

Pictures were taken with Canon EOS 7D Mark II camera attached to an Olympus SZX16 microscope. Focusing and camera were controlled by Deep Focus module for QuickPHOTO 3.1 (Promicra). Focus stacking of the pictures was done by CombineZP (available at http://combinezp.software.informer.com/download/). Specimens were kept in ethanol while photographed.

### 2.3 DNA extraction

We gathered samples from multiple Cimicidae species for our phylogenetic analysis as detailed in Table 1. DNA was extracted from the whole specimen in the case of the *Cimex* sp. samples, or legs in the case of the fresh *B. chiloensis* and museum *P. cavernis* specimen. DNA was extracted using the NucleoSpin® Tissue Kit (product nr 740,952, Macherey‐Nagel), according to the instructions for standard protocol (User manual, version June 2014/Rev. 14) provided with the kit. The *P. cavernis *museum samples were cleaned before the extraction to remove all the non‐target material from the sample surface as follows: (a) samples were vortexed briefly in a tube containing 2% bleach and incubated for 10 min, (b) bleach was removed and samples were washed by adding 99% ethanol, and then (c) rinsed with dd‐H_2_O and finally dried, and then extracted as above. The laboratory and the equipment were sterilized before each extraction batch.

### 2.4 PCR and sequencing

For each extract, we amplified five genes, both nuclear and mitochondrial, using primers and protocols after Balvin et al. (2015). Shortly, the cytochrome oxidase subunit I (COI) was amplified using Lep1Fdeg/Lep3R (Hajibabaei, Janzen, Burns, Hallwachs, & Hebert, 2006), 16S ribosomal gene (16S) using 16S_LR‐J (Kambhampati & Smith, 1995)/16S_LR‐N (Simon et al., 1994), 18S ribosomal gene (18S) in two overlapping fragments: 18S‐1/18S‐3 and 18S‐2/18S‐4 (Tian, Zhu, Li, Xie, & Bu, 2008), Internal transcribed spacer (ITS2) using CAS5p8sFc/CAS28sB1d (Kim & Lee, 2008), and finally Elongation factor 1 subunit α (EF1a) with rcM52.6 (also known as Shirley; Cho et al., 1995)/M2412 (also known as Prowler; Damgaard, Andersen, & Sperling, 2000). For old museum sample *P. cavernis*, we first tried LCO1490/HCO2198 (Folmer, Black, Hoeh, Lutz, & Vrijenhoek, 1994) which failed to yield results, and subsequently received product with LCO1490 with C_R (Shokralla et al., 2015). The following PCR setup was used for all samples: 2 µl of the template DNA was mixed with 300 nM of each primer, 5 µl of 2× MyTaq RedMix (Bioline) and the reaction was filled up to 10 µl with double‐distilled water. The PCR cycling conditions were as follows: initial denaturation for 5 min in 95°C, then 35 cycles of denaturation for 30 s in 95°C, annealing for 30 s in 42–57°C (the annealing temperature was gene‐specific as detailed in Balvín et al. (2015), and elongation for 30 s in 72°C, ending with final elongation step for 5 min in 72°C. A blank control was included in each PCR batch. For the *P. cavernis* sample, we tried to increase PCR success by adding more DNA (up to 6 µl), by increasing the total volume (up to 20 µl), and by increasing the number of PCR cycles to 50. For all genes, successful PCR products were cleaned by adding 1 µl of Exonuclease I and 1.0 µl of FastAP (both included in the A'SAP clean kit; product nr 80350, ArcticZymes, Trømssa, Norway) to each product, and by heating the mix to 37°C for 10 min and 85°C for 5 min. After that, sequences were shipped to Macrogen Europe (Macrogen, Seoul, Rep. of Korea) for sequencing. Resulting sequences were trimmed for sequencing primers and non‐reliable poor‐quality regions and then aligned per gene using Geneious R6 (Kearse et al., 2012).

### 2.5 Phylogenetic analysis

To construct Cimicidae phylogeny, we downloaded all sequences used by Balvín et al. (2015). These included sequences from *Cimex pipistrelle *Jenyns, 1839, *C. adjunctus *Barber, 1939, *C. japonicus* Usinger, 1966, *C. hemipterus *Fabricius, 1803, *C. lectularius *L., Cimex sp., *C. latipennis *Usinger & Ueshima, 1965, *C. pilosellus*, *C.* cf. *antennatu*s, *Cacodmus vicinus *Horvath, 1934, *Cacodminae *sp., *Oeciacus vicarius *Horvath, 1912, *O. hirundinis *(Lamarck, 1816), *Paracimex setosus *Ferris & Usinger, 1957, *Aphrania elongata *Usinger, 1966. Additionally, we downloaded sequences of *Leptocimex inordinatus *Ueshima, 1968 from GenBank. Similarly, for phylogenetic outgroup, we retrieved Cimicomorpha sequences from *Rhodnius prolixus* Stål, 1859 (Reduviidae), *Lygus elisus *Van Duzee, 1914 (Miridae), and *Orius niger* (Wolff, 1811) (Anthocoridae) following Balvín et al. (2015). The accession codes are listed in Table 1. Unfortunately, despite rather comprehensive data set, we could not retrieve fresh samples or sequences for all the Cimicidae species found in South America, for example those collected from Argentina (Di Benedetto, Autino, González, & Argoitia, 2017). All the samples with accession codes and other metadata are collected in Table 1.

For the sequences produced in this study, the primers and low quality regions were trimmed of the sequences, and all the sequences including references from GenBank were aligned with MUSCLE plugin (Edgar, 2004) using software Geneious (Kearse et al., 2012). First, we used GenBank BLAST analysis to check whether our trimmed sequences were free from contamination. For some of the samples, only COI sequences were available, so we prepared two different data sets: 1 (multilocus: 52‐taxon set) and 2 (COI: 56‐taxon set). See Table 1 for details of samples in each data set. For these two data sets, two model‐based methods (Bayesian inference and maximum likelihood) were used to analyze the data.

Bayesian phylogenetic analyses were carried out using the program MrBayes v3.2.3 ×64 (Huelsenbeck & Ronquist, 2001) in CSC servers (www.csc.fi). The GTR+G (with four rate categories for Gamma) model of substitution was fitted to each data set. The data sets were subjected to two runs of one million generations each, with every 1000th generation sampled and the first 2,500 sampled generations discarded as burn‐in. Similarly, we constructed a maximum‐likelihood tree with 100 bootstrap replicates (other settings as default) using command line PhyML (version 20120412) (Guindon & Gascuel, 2003) at CSC servers. The posterior probability tree from Bayesian analysis and consensus tree from ML was retrieved and imported to Geneious to draw the final tree.

## 3 RESULTS

### 3.1 Morphological characters in the Primicinae

**Figure 3:**
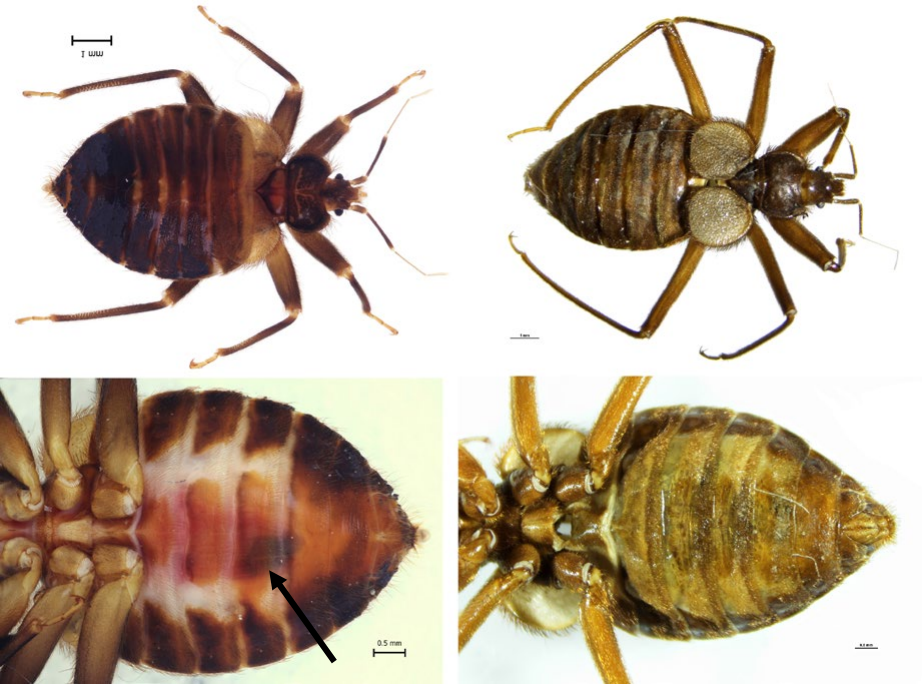
Dorsal and ventral views of *Bucimex chilensis* (left) and *Primicimex cavernis* (right). Black arrow indicates spermalage on *B. chilensis*, which is missing from *P. cavernis*

**Figure 4:**
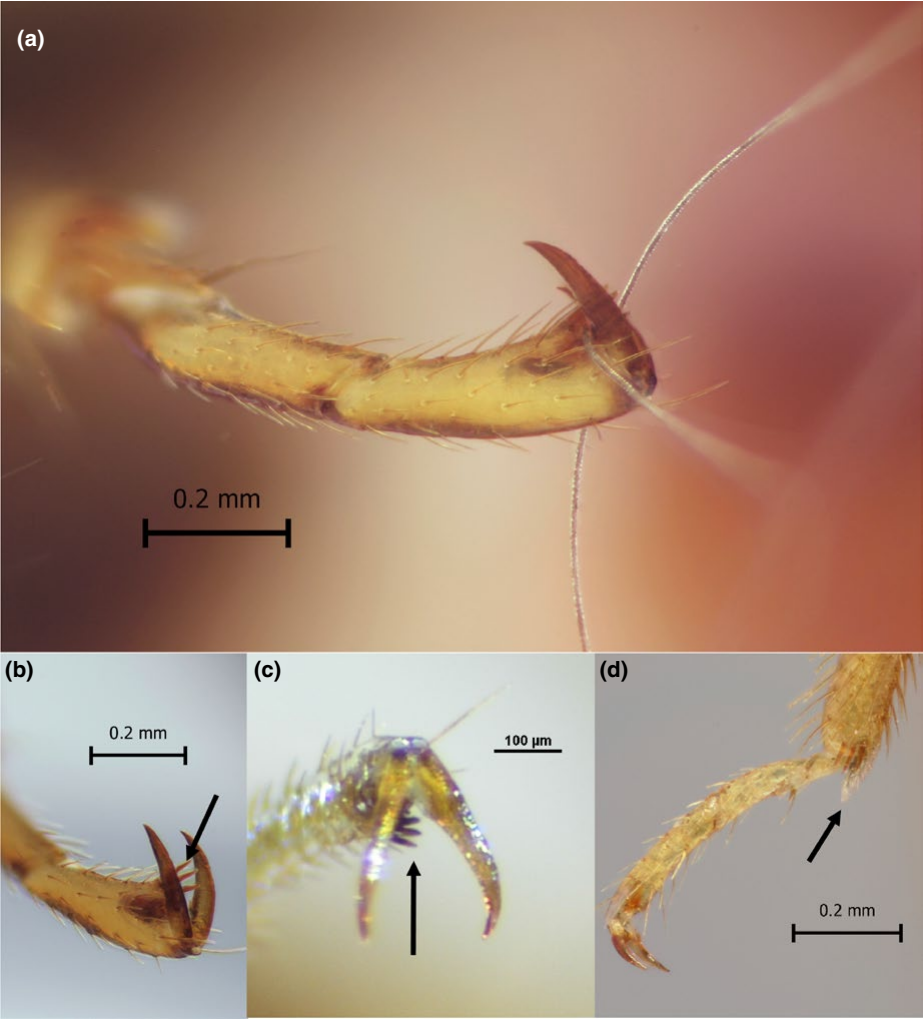
(a) Tarsal claws clinging on to *Myotis chiloensis* fur. (b) Tarsal claws and erect ctenidea (black arrow), which facilitate grasping host hair. (c) A similar tarsal structure on *Primicimex cavernis* with ctenidea (black arrow). (d) The tibia and tarsi of *Cimex lectulariarus* with specialized setae on the joint (black arrow), which may be used to fasten the bug to the plagiopatagium of the bat

The Primicimicinae individuals obtained were morphologically identified as *B. chilensis*, *P. cavernis* (Figure 3). The subfamily Primicimicinae, to which both species examined belong to, differs from other Cimicidae by having mottled tibiae (Figures 3 and 4a,b,c), labrum over twice as long as wide and tarsi with several erect ctenidea (spines) at inner apex in apposition to claws (Figure 4a,b,c), for which the ecological function has not been suggested prior to this study. The two Primicimicinae species are similar in appearance, but may be separated by the relative length of femora and the length of first antennal segment, which is as long as the second segment in *P. cavernis* and much shorter in *B. chilensis*. *Primicimex cavernis* lacks the mycetomes and spermalege found in other cimicid groups. *Bucimex chilensis*, on the other hand, exhibits mycetomes and a well‐developed spermalege (Figure 3). The Primicimicinae tarsi differ significantly from the tarsi of *C. lectularius* (Figure 4d), which lack the erect ctenidea at the inner apex in apposition to claws. In addition to this, the *C. lectularius* feature additional spines and a spike at the joint between the tibia and tarsus. The other species in this study were morphologically identified to *C. lectularius *(Finnish specimen) or *Cimex* sp. (Canadian specimen).

### 3.2 Molecular identification of the specimens

In the BLAST analysis, the closest match for *B. chilensis* COI sequence in GenBank was a record of *Orius minutus* (Linnaeus, 1758) (Hemiptera, Cimicoidae, Anthocoridae) with very low similarity (82%, E‐value 7e‐151; BLAST was performed online 22nd March 2017). For *P. cavernis*, we were only able to retrieve 309 bp sequence of COI from the type specimens in this analysis. For this sequence, the closest match (83%, E‐value 1e‐68) was to *Liorhyssus hyalinus* (Fabricius, 1794) (Hemiptera, Coreoidea, Rhopalidae). The percentage identity between query and subject sequence was naturally too low to make any conclusions about phylogenetic relationships based on the BLAST analysis. Finnish samples were molecularly confirmed as *C. lectularius*, and the Canadian samples were confirmed to include both *C. pilosellus* and *C. adjunctus*. All the sequences produced in this study were uploaded to GenBank with accession codes MK141690–MK141706.

### 3.3 Phylogenetic analysis of the Cimicidae

**Figure 5:**
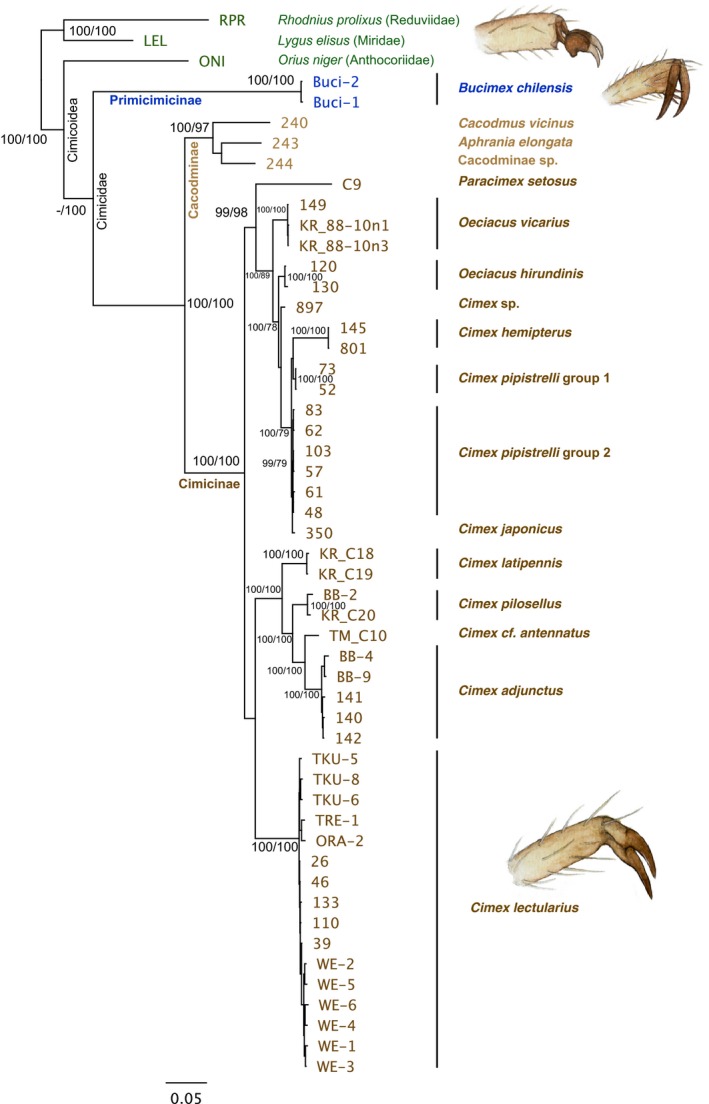
A multilocus DNA‐based phylogeny of Cimicidea using five genes with support values for all the main clades based on both Bayesian posterior probability (left number) and maximum‐likelihood analysis with 100 bootstrap replicates (right). Three Cimicomorpha families outside Cimicidae are used as an outgroup to root the tree. The higher taxa within Cimicoidea are marked in the clades. The morphological differences in the tarsi are illustrated for comparison: *Orius niger* (representing Anthocoridae and other outgroups), *Bucimex chilensis *(Primiciminae), and *Cimex lectularius* (Cimicinae+Cacodminae)

**Figure 6:**
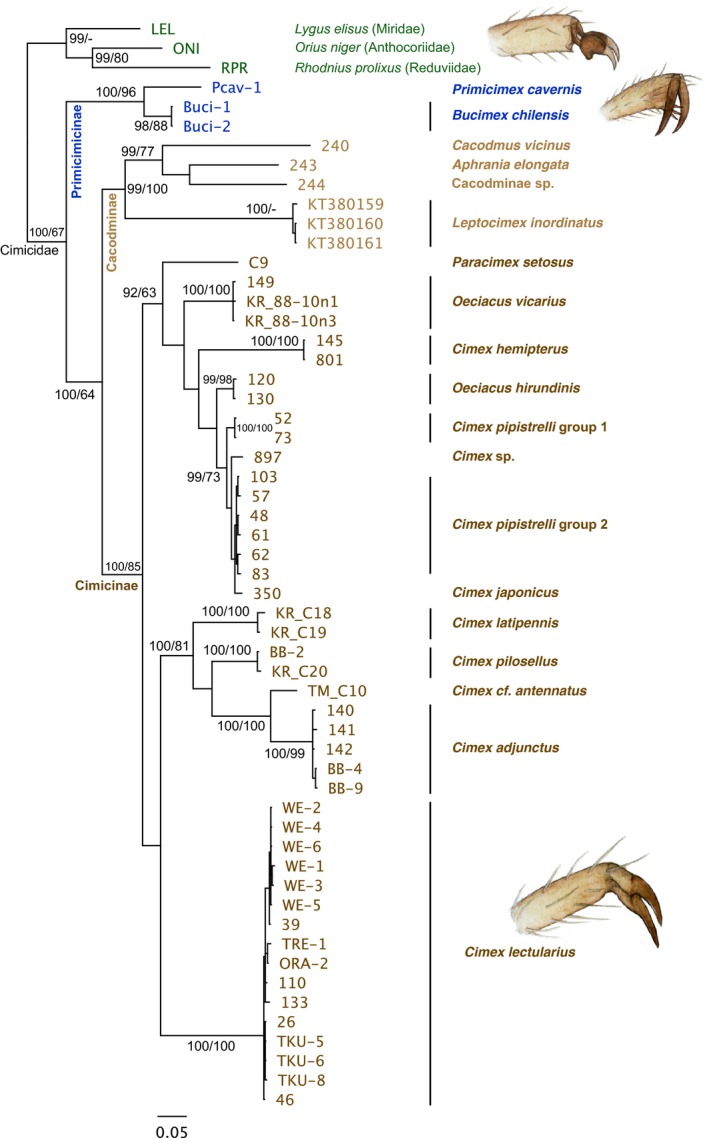
A phylogeny of Cimicidea using COI gene with support values for all the main clades based on both Bayesian posterior probability (left number) and maximum‐likelihood analysis with 100 bootstrap replicates (right). Both Primiciminae species (*B. chilensis* and *P. cavernis*) cluster close to each other at the base of Cimicidae with high support. The subfamily Cacodminae is also strongly supported, but subfamily Cimicinae is poorly resolved, such as polyphyletic genus *Cimex*

The multilocus analysis of five genes using 52 taxa (in data set 1; Table 1) placed the *B. chilensis* samples into the base of the Cimicidae, next to the outgroup families Anthocoriidae (Cimicoidea), Reduviidae, and Miridae (Figure 5). The same patterns occurred in both Bayesian and ML trees (Figure 5). Moreover, the COI phylogenetic analysis using 56 taxa (in data set 2; Table 1) produced almost identical patterns compared to the multilocus tree (Figure 6). In COI tree, the *B. chilensis* and *P. cavernis* cluster close to each other forming an own clade at the base of Cimicidae (Figure 6). Subfamily Cacodminae appears to be a monophyletic group, and genus *Leptocimex* is confirmed as a member of Cacodminae (Figure 6). On the other hand, the genus *Oeciacus* appears to be paraphyletic, the two species not clustering together, and furthermore, the *Cimex pipistrelli* splits into two distinct groups together with *C. japonicus* and *C. sp*. All *C. lectularius* specimens, the “bed bug,” cluster in the same clade regardless of geographical origin (Figures 5 and 6). The other *Cimex* species (*hemipterus, pilosellus, cf. antennatus*, and *adjunctus*) form separate clusters in all analysis (Figures 5 and 6). In both multilocus and COI phylogenies, the genus *Cimex* seems to be polyphyletic, despite high support for all species groupings (Figures 5 and 6).

## 4 DISCUSSION

For the first time, we place the two known taxa in subfamily Primicimicinae into a molecular phylogeny of Cimicidae. In our phylogeny, Primicimicinae is a sister group to all other Cimicidae, as described morphologically by Usinger (1966). We also found that the two members of the Primiciminae subfamily have unique tarsal morphology (ctenidium) for attaching to their bat hosts, and that these traits may be ancestral among the cimicids. Cimicids in the *C. pipistrellus *groups and *C. lectularius* are often loosely attached to the wings, forearms and uropatagium of the bat (Balvín, Sevcik, et al., 2012; Heise, 1988). However, the morphology of *B. chilensis* tarsi is very different to other cimicids. The structure of the *B. chilensis *tarsus appears to be an adaptation to clinging on to pelage of the host, which is also where both individuals were found: an atypical location for other cimicids. This “cling‐on” behavior, and the resemblance of the abdomen to the head of the fierce extra‐terrestrial warrior species in the popular TV‐show, Star Trek, suggest the descriptive nickname, “The Klingon batbug.”.

The *B. chilensis* individual was located on dorsal surface of the bat, grasping on to the pelage of the host (Figure 1). After the removal of the cimicid, we observed bat hair clamped in between the tarsal claws and the erect ctenidium between them, displaying the mechanism in action (Figure 4a). This same morphological characteristic is also featured on the *P. cavernis*, suggesting it may also spend extended periods on the host. This feature, absent in other cimicids, appears to be only shared by the two members of the subfamily Primicimicinae. A similar functional adaptation has evolved in the family Polyctenidae (Heteroptera), which have several functional morphological adaptations that facilitate the obligate association with bats and continuous living on the host specimen: for example, lack of wings, short antennae and most importantly, in comparison with *Bucimex chilensis*, form of tibiae, claws and associated erect ctenidea (Maa, 1964). In addition, the tarsal claws have been proposed to be the most important structure for host attachment also in bat flies (Dick & Patterson, 2006). This behavior is very different to more many other cimicids, which, for their limited dispersal, use a very different mechanism to attach to the plagiopatagium or tibia of the bat as depicted in Balvín, Sevcik, et al., 2012). This mode of attachment may be facilitated by the specialized setae located apicoventrally on the tibia, as well as the stiff spines, which flank the tibia and tarsus joint (Figure 3d).

Before the discovery of our sample, individuals of *Bucimex chilensis *described in Usinger (1966) had been obtained from Chile from *Araucaria araucana* (Molina) K. Koch trees at Tolhuaca (38°S, 71°W); in a *Nothofagus *sp*.* hollow in Lonquimay (38°S, 71°W), Araucanía region and in *Nothofagus *sp. at Dalcahue (42°S, 73°W), Los Lagos region, associated with either *M. chiloensis* or *Histiotus magellanicus* Philippi, 1866 bat colonies (Usinger, 1966) (Figure 2). Our new record in Tierra del Fuego is 1,300 km to the south (58°S, 69°W) of this previous record. Because *M. chiloensis* is not a migrating species (Rodriguez‐San Pedro, Allendes, & Ossa, 2016), the new geographical record is most likely not due to a range expansion, but rather reflects the lack of research on bats or the associated invertebrates in the southern latitudes. As for the northern range, the *B. chilensis* has not been reported from northern Argentina (Autino, Claps, Sanchez, & Barquez, 2009), but the distribution most likely extends further north of 38°S in Chile.

The results of the molecular phylogenetic analysis using a multilocus approach are in accord with the previous phylogenies based on morphology and molecular data (Balvín et al., 2015; Hornok et al., 2017; Li et al., 2012; Reinhardt & Siva‐Jothy, 2007; Usinger, 1966), with the addition of Primicimicinae and associated taxa added. The single locus results, using the COI gene and including the *P. cavernis* (and *Leptocimex inordinatus*) samples, of which only this single gene was retrieved, also reflected the aforementioned studies. Both phylogenies constructed in this study place *P. cavernis* next to the *B. chilensis*, indicating a strong phylogenetic signal. To further strengthen our findings, these two species share many primitive features, such as the structure of the male and female genitals, as well as features that are specific to the subfamily Primiciminae, such as the tarsal structure and mottled tibiae, which are absent in other cimicids. Therefore, the subfamily status of Primiciminae is supported. However, the missing spermalege of *P. cavernis*, which Usinger (1966) regards as a primitive trait may rather be a derived one (Reinhardt & Siva‐Jothy, 2007), because the spermalege is present in Anthocoridae, the sister group of Cimicidae (Hangay et al., 2008). Although the close relatedness between *Bucimex* and *Primicimex* seems to be robust. Clearly, more work is needed to fully resolve the phylogenetic relationships within family Cimicidae (especially the paraphyly of Oeciacus), superfamily Cimicoidea, and the whole infraorder Cimicomorpha.

The dispersal of individuals between roosts is crucial in maintaining local and range wide genetic diversity of bat bugs, but also allows invasion of new or temporarily abandoned roosts. However, this appears to happen rather infrequently, with only 3% of surveyed *Nyctalus noctula* (Schreber, 1774) carrying *C. pipistrellus* bugs in a large study by Heise, (1988). The predominance of adult female *Cimex* found on bat hosts in the outside roost environment supports the idea that remaining attached to the host is deliberate and serves the purpose of dispersal (Balvín, Sevcik, et al., 2012; Heise, 1988). Because a single‐mated female has the ability to initiate a new infestation, they are the most effective agents of dispersal (Bartonicka & Gaisler, 2007; Usinger, 1966). This may also be true for *P. cavernis*, for which habitat, host choice, and feeding behavior have been described in detail by Ueshima (1968). The tarsal structures most likely facilitate dispersal in this cave‐dwelling species, which has access to thousands of hosts. However, Tierra del Fuego is cave‐free and population density of hosts is low. One of the radiotrackedhost individuals (*M. chiloensis*) in this study appeared to roost solitary in a hollow tree, which is a relatively unsheltered roost with fluctuating climatic conditions. An individual tree can only be considered a semi‐permanent roost, often only used by bats for some years, when trees are at a certain degree of decay (Lacki & Baker, 2003; Lacki, Baker, & Johnson, 2012). Tree‐roosting bats also use several roosts within their home range and show a high degree of roost switching within a season (Kerth, Ebert, & Schmidtke, 2006; Lewis, 1995).Therefore, a low host population density and temporary use of roosts by the hosts may necessitate a more permanent, ectoparasitic life‐history for *B. chilensis* attached to its host.

Most cimicids are generalist when it comes to host species choice, although host association can influence variation in salivary gene proteins in populations specializing in specific host species (Talbot, Vonhof, Broders, Fenton, & Keyghobadi, 2018). Both *C. lectularius* and *C. pipistrellii* have been described from many bat host species (Balvin et al., 2014). *Primicimex cavernis* has been described as expressing host specificity toward *Tadarida brasiliensis* (Ueshima, 1968). Ney cave in, Medina Co. Texas, where the species has been described from (Usinger, 1966), is a seasonal roost for *T. brasiliensis* as well as *Mormoops megalophylla*, which may act as a secondary host (M. Meierhofer, pers. comm.). So far, *B. chilensis* has been described on *M. chiloensis* and *Histiotus magellanicus *(Usinger, 1966). Convergent phenotypes in ectoparasites can often be seen among different lineages of a higher taxon or even within a single species (McCoy et al., 2005). For instance, *C. lectularius* and *C. pipistrellus*, have been found to be an interesting model for the study of within‐species morphological diversification (Balvín, Munclinger, et al., 2012). The development of convergent phenotypes, or in an extreme situation, alloxenic speciation, could be mediated by reproductive barriers, which are likely associated with local adaptation of the parasite and shift in its host specificity (Poulin, 2007). Both bat species known to host *B. chilensis* use trees and buildings to form their colonies (Mann, 1978), and may be shared between the species, but the colonies of *H. magellanicus* are smaller than colonies of *M. chiloensis* in southern Chile. Altamirano et al (2017) described the use of tree holes of *H. magellanicus* at the Araucanía region, showing that the colonies were formed by no more than 10 adult individuals and they change roost frequently during the year (Altamirano et al., 2017). On the other hand, when buildings are used, colonies of *M. chiloensis* can consist of hundreds of individuals (Ossa et al., 2010). However, we have yet to observe *B. chilensis* on *H. magellanicus* or any other bat species. Further elucidating the host specificity and ecology of *B. chilensis* would require a better understanding of roosting behavior of the host species and acquiring specimens from a variety of host species and geographic areas. For instance, because of its habit of attaching to the pelage of the host, convergent phenotypes on different host species and geographic areas may require morphological changes in the claw structure to facilitate the differences in hair structure.

Here, we describe the Klingon bat bug and its ability to adhere to their host at the southernmost distribution of the species range, 1,300 km to the south of the previously known southernmost distribution boundary in Chile. Our findings show that basal cimicids possess adaptations for grasping on to the pelage of hosts. In contrast, more derived species use setae and spines on the tibia for briefly adhering to the wing of the host. The greater diversity of more derived species within Cimididae, adaptations for attaching to the wing, instead of clinging to the pelage, suggests this method could have yet undiscovered advantages and warrants further investigation. Our results are mostly coincident with previous phylogenies based on morphology. Because of the difficulties in obtaining cimicid specimens from austral South America, this study fills a gap in the knowledge of this cryptic parasite‐host relationship.

## AUTHOR CONTRIBUTIONS

TML, GO, and JSJ designed the study, conducted the field work, and produced the first draft of the manuscript. AP and EJV conducted the laboratory work, sequenced the samples, and analyzed the data. VR and IES contributed to systematics and taxonomy. All authors contributed to the final version of the manuscript.

## Data Availability

All the sequences produced in this study were uploaded to GenBank with accession codes as specified in Table 1.
